# The Complete Chloroplast Genome Sequences of the Medicinal Plant *Forsythia suspensa* (Oleaceae)

**DOI:** 10.3390/ijms18112288

**Published:** 2017-10-31

**Authors:** Wenbin Wang, Huan Yu, Jiahui Wang, Wanjun Lei, Jianhua Gao, Xiangpo Qiu, Jinsheng Wang

**Affiliations:** 1 College of Life Science, Shanxi Agricultural University, Taigu 030801, China; sxndyh@stu.sxau.edu.cn (H.Y.); leiwanjunleo@sxau.edu.cn (W.L.); gaojh_edu@sxau.edu.cn (J.G.); sxndqiuxiangpo@stu.sxau.edu.cn (X.Q.); 2 College of Plant Protection, Northwest Agriculture & Forestry University, Yangling 712100, China; wjh1014@nwafu.edu.cn

**Keywords:** *Forsythia suspensa*, sequencing, chloroplast genome, comparative genomics, phylogenetic analysis

## Abstract

*Forsythia suspensa* is an important medicinal plant and traditionally applied for the treatment of inflammation, pyrexia, gonorrhea, diabetes, and so on. However, there is limited sequence and genomic information available for *F. suspensa*. Here, we produced the complete chloroplast genomes of *F. suspensa* using Illumina sequencing technology. *F. suspensa* is the first sequenced member within the genus *Forsythia* (Oleaceae). The gene order and organization of the chloroplast genome of *F. suspensa* are similar to other Oleaceae chloroplast genomes. The *F. suspensa* chloroplast genome is 156,404 bp in length, exhibits a conserved quadripartite structure with a large single-copy (LSC; 87,159 bp) region, and a small single-copy (SSC; 17,811 bp) region interspersed between inverted repeat (IRa/b; 25,717 bp) regions. A total of 114 unique genes were annotated, including 80 protein-coding genes, 30 tRNA, and four rRNA. The low GC content (37.8%) and codon usage bias for A- or T-ending codons may largely affect gene codon usage. Sequence analysis identified a total of 26 forward repeats, 23 palindrome repeats with lengths >30 bp (identity > 90%), and 54 simple sequence repeats (SSRs) with an average rate of 0.35 SSRs/kb. We predicted 52 RNA editing sites in the chloroplast of *F. suspensa*, all for C-to-U transitions. IR expansion or contraction and the divergent regions were analyzed among several species including the reported *F. suspensa* in this study. Phylogenetic analysis based on whole-plastome revealed that *F. suspensa*, as a member of the Oleaceae family, diverged relatively early from Lamiales. This study will contribute to strengthening medicinal resource conservation, molecular phylogenetic, and genetic engineering research investigations of this species.

## 1. Introduction

*Forsythia suspensa* (Thunb.) Vahl, known as “Lianqiao” in Chinese, is a well-known traditional Asian medicine that is widely distributed in many Asian and European countries [1]. In folk medicine, the extract of the dried fruit has long been used to treat a variety of diseases, such as inflammation, pyrexia, gonorrhea, tonsillitis, and ulcers [2]. In recent years, the dried ripe fruit of *F. suspensa* has often been prescribed for the treatment of diabetes in China [3,4].

Chloroplast (cp) genomes are mostly circular DNA molecules, which have a typical quadripartite structure composed of a large single copy (LSC) region and a small single copy (SSC) region interspersed between two copies of inverted repeats (IRa/b) [5]. The cp genome sequences can provide vast information not only about genes and their encoded proteins, but also on functional implications and evolutionary relationships [6]. Due to high-throughput capabilities and relatively low costs, next-generation sequencing techniques have made it more convenient to obtain a large number of cp genome sequences [7]. After the first complete cp DNA sequences were reported in *Nicotiana tabacum* [8] and *Marchantia polymorpha* [9], complete cp DNA sequences of numerous plant species were determined [6,10,11,12]. To date, approximately 1300 plant cp genomes are publicly available as part of the National Center for Biotechnology Information (NCBI) database. 

Within the Oleaceae family, the complete cp genomes of several plant species have been published [12,13,14,15], thereby providing additional evidence for the evolution and conservation of cp genomes. Nevertheless, no cp genome belonging to genus *Forsythia* has been reported. Few data are available with respect to the *F. suspensa* cp genome. 

In order to characterize the complete cp genome sequence of the *F. suspensa* and expand our understanding of the diversity of the genus *Forsythia*, details of the cp genome structure and organization are reported in this paper. This is also the first sequenced member of the genus *Forsythia* (Oleaceae). We compare the *F. suspense* cp genome with previously annotated cp genomes of other Lamiales species. Our studies could provide basic data for the medicinal species conservation and molecular phylogenetic research of the genus *Forsythia* and Lamiales.

## 2. Results and Discussions

### 2.1. Genome Features

Whole genome sequencing using an Illumina Hiseq 4000 PE150 platform generated 19,241,634 raw reads. Clean reads were obtained by removing adaptors and low-quality read pairs. Then, we collected 662,793 cp-genome-related reads (3.44% of total reads), reaching an average of 636 × coverage over the cp genome. With PCR-based experiments, we closed the gaps and validated the sequence assembly, and ultimately obtained a complete *F. suspensa* cp genome sequence, which was then submitted to GenBank (accession number: MF579702).

**Figure 1:**
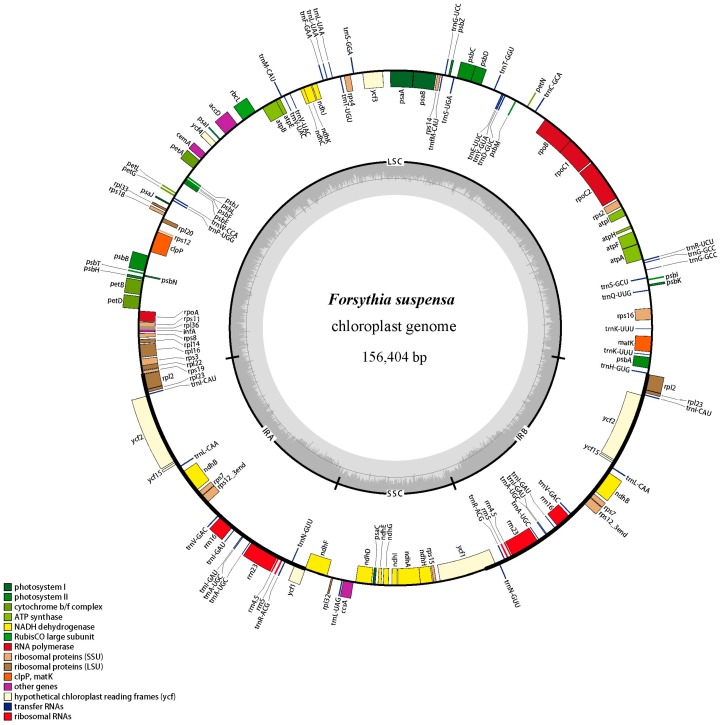
Chloroplast genome map of *Forsythia suspensa*. Genes drawn inside the circle are transcribed clockwise, and those outside are counterclockwise. Genes are color-coded based on their function, which are shown at the left bottom. The inner circle indicates the inverted boundaries and GC content.

Most cp genomes of higher plants have been found to have a typical quadripartite structure composed of an LSC region and an SSC region interspersed between the IRa/b region [5]. The complete cp genome of *F. suspensa* has a total length of 156,404 bp, with a pair of IRs of 25,717 bp that separate an LSC region of 87,159 bp and an SSC region of 17,811 bp (Figure 1). The total GC content was 37.8%, which was similar to the published Oleaceae cp genomes [12,13,14,15]. The GC content of the IR regions was 43.2%, which was higher when compared with the GC content in the LSC and SSC regions (35.8% and 31.8%, respectively). 

The gene content and sequence of the *F. suspensa* cp genome are relatively conserved, with basic characteristics of land plant cp genomes [16]. It encodes a total of 114 unique genes, of which 19 are duplicated in the IR regions. Out of the 114 genes, there are 80 protein-coding genes (70.2%), 30 tRNA (26.3%), and four rRNA genes (*rrn5*, *rrn4.5*, *rrn16*, *rrn23*) (3.5%) (Table 1). Eighteen genes contained introns, fifteen (nine protein-coding and six tRNA genes) of which contained one intron and three of which (*rps12*, *ycf3*, and *clpP*) contained two introns (Table 2). The *rps12* gene is a trans-spliced gene, three exons of which were located in the LSC region and IR regions, respectively. The complete gene of *matK* was located within the intron of *trnK-UUU*. One pseudogene (non functioning duplications of functional genes), *ycf1*, was identified, located in the boundary regions between IRb/SSC. The partial gene duplication might have caused the lack of protein-coding ability. In general, the junctions between the IR and LSC/SSC regions vary among higher plant cp genomes [17,18,19]. In the *F. suspensa* cp genome, the *ycf1* gene regions extended into the IR region in the IR/SSC junctions, while the *rpl2* was 51 bp apart from the LSC/IR junction.

### 2.2. Comparison to Other Lamiales Species

**Figure 2:**
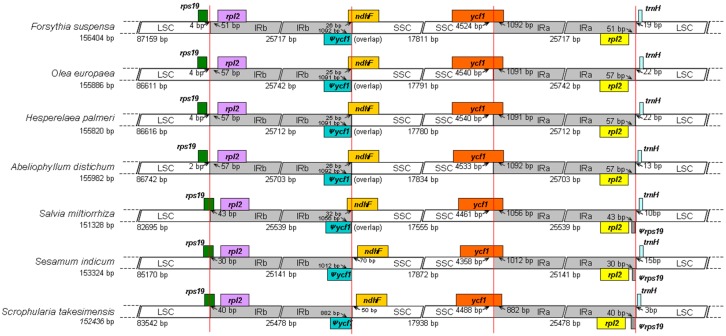
Comparisons of LSC, SSC, and IR region borders among six Lamiales chloroplast genomes. *Ψ* indicates a pseudogene. Colorcoding mean different genes on both sides of the junctions. Number above the gene features means the distance between the ends of genes and the junction sites. The arrows indicated the location of the distance. This figure is not to scale.

The IR regions are highly conserved and play an important role in stabilizing the cp genome structure [20,21]. For IR and SC boundary regions, their expansion and contraction are commonly considered as the main mechanism behind the length variation of angiosperm cp genomes [22,23]. In this study, we compared the junctions of LSC/IRb/SSC/IRa of the seven Lamiales cp genomes (Figure 2), and also observed the expansions and contractions in IR boundary regions. 

The *rps19* genes of four Oleaceae species were all completely located in the LSC region, and the IR region expanded to the *rps19* gene in the other three genomes, with a short *rps19* pseudogene of 43 bp, 30 bp, and 40 bp created at the IRa/LSC border in *S. miltiorrhiza*, *S. indicum*, and *S. takesimensis*, respectively. The border between the IRb and SSC extended into the *ycf1* genes, with *ycf1* pseudogenes created in all of the seven species. The length of the *ycf1* pseudogene was very similar in four of the Oleaceae species (1091 or 1092 bp), and was longer than that in *S. miltiorrhiza* (1056 bp), *S. indicum* (1012 bp), and *S. takesimensis* (886 bp). Overlaps were detected between the *ycf1* pseudogene and the *ndhF* gene in five cp genomes (except for *S. indicum* and *S. takesimensis*), which also had similar lengths (25 or 26 bp) in four Oleaceae species. The *trnH-GUG* genes were all located in the LSC region, the distance of which from the LSC/IRa boundary was 3–22 bp. Overall, the IR/SC junctions of the Oleaceae species were similar and showed some difference compared to those of Lamiaceae (*S. miltiorrhiza*), Pedaliaceae (*S. indicum*), and Scrophulariaceae (*S. takesimensis*). Our results suggested that the cp genomes of closely related species might be conserved, whereas greater diversity might occur among species belonging to different families, such as one inverted repeat loss in the cp genome of *Astragalus membranaceus* [24] and the large inversions in *Eucommia ulmoides* [25].

### 2.3. Codon Usage Analysis

The synonymous codons often have different usage frequencies in plant genomes, which was termed codon usage bias. A variety of evolutionary factors which affect gene mutation and selection may lead to the occurrence of codon bias [26,27]. 

To examine codon usage, the effective number of codons (Nc) of 52 protein-coding genes (PCGs) was calculated. The Nc values for each PCG in *F. suspensa* are shown in Table S2. Our results indicated that the Nc values ranged from 37.83 (*rps14*) to 54.75 (*ycf3*) in all the selected PCGs. Most Nc values were greater than 44, which suggested a weak gene codon bias in the *F. suspensa* cp genome. The *rps14* gene was detected to exist in the most biased codon usage with the lowest mean Nc value of 37.83. Table 3 showed the codon usage and relative synonymous codon usage (RSCU). Due to the RSCU values of >1, thirty codons showed the codon usage bias in the *F. suspensa* cp genes. Interestingly, out of the above 30 codons, twenty-nine were A or T-ending codons. Conversely, the G + C-ending codons exhibited the opposite pattern (RSCU values < 1), indicating that they are less common in *F. suspensa* cp genes. Stop codon usage was found to be biased toward TAA. The similar codon usage rules of bias for A- or T-ending were also found in poplar, rice, and other plants [28,29,30].

The factors affecting codon usage may vary in different genes or species. In a relative study, Zhou et al. [30] considered the genomic nucleotide mutation bias as a main cause of codon bias in seed plants such as arabidopsis and poplar. Morton [31] reported that the cp gene codon usage was largely affected by the asymmetric mutation of cp DNA in *Euglena gracilis*. Our result suggested that a low GC content and codon usage bias for A + T-ending may be a major factor in the cp gene codon usage of *F. suspensa*.

The 52 unique PCGs comprised 63,555 bp that encoded 21,185 codons. The amino acid (AA) frequencies of the *F. suspensa* cp genome were further computed. Of these codons, 2237 (10.56%) encode leucine, which was the most frequency used AA in the *F. suspensa* cp genome (Table 3). As the least common one, cysteine was only encoded by 223 (1.05%) codons. 

### 2.4. Repeats and Simple Sequence Repeats Analysis

**Figure 3:**
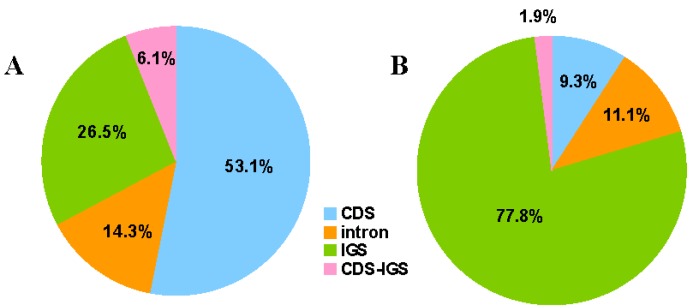
Distribution of repeat sequence and simple sequence repeats (SSRs) within *F. suspensa* chloroplast genomes. (**A**) Distribution of repeats; and (**B**) distribution of SSRs. IGS: intergenic spacer.

Repeat sequences in the *F. suspensa* cp genome were analyzed by REPuter and the results showed that there were no complement repeats and reverse repeats. Twenty-six forward repeats and 23 palindrome repeats were detected with lengths ≥ 30 bp (identity > 90%) (Table 4). Out of the 49 repeats, 34 repeats (69.4%) were 30–39 bp long, 11 repeats (22.4%) were 40–49 bp long, four repeats (8.2%) were 50–59 bp long, and the longest repeat was 58 bp. Generally, repeats were mostly distributed in noncoding regions [32,33]; however, 53.1% of the repeats in the *F. suspensa* cp genome were located in coding regions (CDS) (Figure 3A), mainly in *ycf2*; similar to that of *S. dentata* and *S. takesimensis* [34]. Meanwhile, 40.8% of repeats were located in intergenic spacers (IGS) and introns, and 6.1% of repeats were in parts of the IGS and CDS.

Simple sequence repeats (SSRs) are widely distributed across the entire genome and exert significant influence on genome recombination and rearrangement [35]. As valuable molecular markers, SSRs have been used in polymorphism investigations and population genetics [36,37]. The occurrence, type, and distribution of SSRs were analyzed in the *F. suspensa* cp genome. In total, we detected 54 SSRs in the *F. suspensa* cp genome (Table 5), accounting for 700 bp of the total sequence (0.45%). The majority of these SSRs consisted of mono- and di-nucleotide repeats, which were found 35 and seven times, respectively. Tri-(1), tetra-(4), and penta-nucleotide repeat sequences (1) were detected with a much lower frequency. Six compound SSRs were also found. Fifty SSRs (92.6%) were composed of A and T nucleotides, while tandem G or C repeats were quite rare, which was in concordance with the other research results [38,39]. Out of these SSRs, 42 (88.9%) and six (11.1%) were located in IGS and introns, respectively (Figure 3B). Only five SSRs were found in the coding genes, including *rpoC2*, *rpoA*, and *ndhD*, and one was located in parts of the IGS and CDS. In addition, we noticed that almost all SSRs were located in LSC, except for (T)19, and no SSRs were detected in the IR region. These SSRs may be developed lineage-specific markers, which might be useful in evolutionary and genetic diversity studies.

### 2.5. Predicted RNA Editing Sites in the F. suspensa Chloroplast Genes

In the *F. suspensa* cp genome, we predicted 52 RNA editing sites, which occurred in 21 genes (Table 6). The *ndhB* gene contained the most editing sites (10), and this finding was consistent with other plants such as rice, maize, and tomato [40,41,42]. Meanwhile, the genes *ndhD* and *rpoB* were predicted to have six editing sites: *matK*, five; ropC2, three; *accD*, *ndhA*, *ndhF*, *ndhG*, and *petB*, two; and one each in *atpA*, *atpF*, *atpI*, *ccsA*, *petG*, *psbE*, *rpl2*, *rpl20*, *rpoA*, *rps2*, and *rps14*. All these editing sites were C-to-U transitions. The editing phenomenon was also commonly found in the chloroplasts and mitochondria of seed plants [43]. The locations of the editing sites in the first, second, and third codons were 14, 38, and 0, respectively. Of the 52 sites, twenty were U_A types, which was similar codon bias to previous studies of RNA editing sites [10,44]. In addition, forty-eight RNA editing events in the *F. suspensa* cp genome led to acid changes for highly hydrophobic residues, such as leucine, isoleucine, valine, tryptophan, and tyrosine. The conversions from serine to leucine were the most frequent transitions. As a form of post-transcriptional regulation of gene expression, the feature has already been revealed by most RNA editing researches [44]. Notably, our results provide additional evidence to support the above conclusion.

### 2.6. Phylogeny Reconstruction of Lamiales Based on Complete Chloroplast Genome Sequences

**Figure 4:**
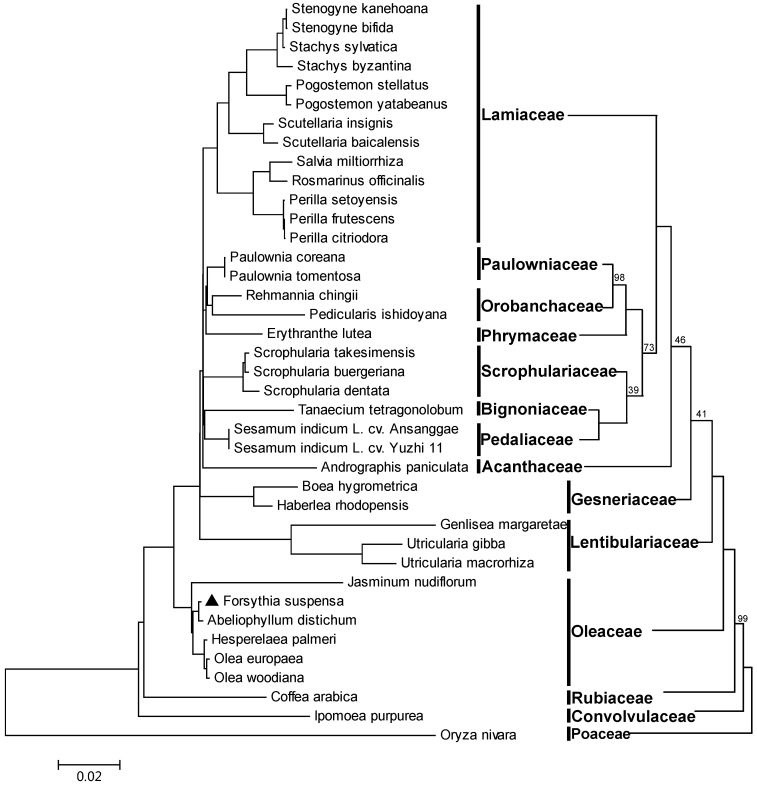
Maximum likelihood phylogeny of the Lamiales species inferred from complete chloroplast genome sequences. Numbers near branches are bootstrap values of 100 pseudo-replicates. The tree on the right panel was constructed manually by reference to the left one, and the distance of branches was meaningless. The branches without numbers indicate 100% bootstrap supports.

Complete cp genomes comprise abundant phylogenetic information, which could be applied to phylogenetic studies of angiosperm [11,45,46]. To identify the evolutionary position of *F. suspensa* within Lamiales, an improved resolution of phylogenetic relationships was achieved by using these whole cp genome sequences of 36 Lamiales species. Three species, *C. Arabica*, *I. purpurea*, and *O. nivara* were also chosen as outgroups. The Maximum likelihood (ML) bootstrap values were fairly high, with values ≥ 98% for 32 of the 36 nodes, and 30 nodes had 100% bootstrap support (Figure 4). *F. suspensa*, whose cp genome was reported in this study, was closely related to *A. distichum*, which then formed a cluster with *H. palmeri*, *J. nudiflorum*, and the Olea species from Oleaceae with 100% bootstrap supports. Notably, Oleaceae diverged relatively early from the Lamiales lineage. In addition, four phylogenetic relationships were only supported by lower ML bootstrap values. This was possibly a result of less samples in these families. The cp genome is also expected to be useful in resolving the deeper branches of the phylogeny, along with the availability of more whole genome sequences.

## 3. Materials and Methods

### 3.1. Plant Materials

Samples of *F. suspensa* were collected in Zezhou County, Shanxi Province, China. The voucher specimens were deposited in the Herbarium of Shanxi Agricultural University, Taigu, China. Additionally, the location of the specimens was not within any protected area.

### 3.2. DNA Library Preparation, Sequencing, and Genome Assembly

Genomic DNA was extracted from fresh young leaves of the *F. suspensa* plant using the mCTAB method [47]. Genomic DNA was fragmented into 400–600 bp using a Covaris M220 Focused-ultrasonicator (Covaris, Woburn, MA, USA). Library preparation was conducted using NEBNext^®^ Ultra™ DNA Library Prep Kit Illumina (New England, Biolabs, Ipswich, MA, USA). Sample sequencing was carried out on an Illumina Hiseq 4000 PE150 platform. 

Next, raw sequence reads were assembled into contigs using SPAdes [48], CLC Genomics Workbench 8 (Available online: http://www.clcbio.com), and SOAPdenovo2 [49], respectively. Chloroplast genome contigs were selected by BLAST (Available online: http://blast.ncbi.nlm.nih.gov/) [50] and were assembled by Sequencher 4.10 (Available online: http://genecodes.com/). All reads were mapped to the cp genome using Geneious 8.1 [51], which verified the selected contigs. The closing of gaps was accomplished by special primer designs, PCR amplification, and Sanger sequencing. Finally, we obtained a high-quality complete *F. suspensa* cp genome, and the result was submitted to NCBI (Accession Number: MF579702).

### 3.3. Genome Annotation and Comparative Genomics

Chloroplast genome annotation was performed using DOGMA (Dual Organellar GenoMe Annotator) [52] (Available online: http://dogma.ccbb.utexas.edue). Putative protein-coding genes, tRNAs, and rRNAs were identified by BLASTX and BLASTN searches (Available online: http://blast.ncbi.nlm.nih.gov/), respectively. The cp genome was drawn using OrganellarGenomeDRAW [53] (Available online: http://ogdraw.mpimp-golm.mpg.de/index.shtml), with subsequent manual editing. The boundaries between the IR and SC regions of *F. suspensa* and six other Lamiales species were compared and analyzed.

### 3.4. Repeat Sequence Analyses

The REPuter program [54] (Available online: https://bibiserv.cebitec. uni-bielefeld.de/reputer) was used to identify repeats including forward, reverse, palindrome, and complement sequences. The length and identity of the repeats were limited to ≥30 bp and >90%, respectively, with the Hamming distance equal to 3 [55,56]. The cp SSRs were detected using MISA [57] with the minimum repeats of mono-, di-, tri-, tetra-, penta-, and hexanucleotides set to 10, 5, 4, 3, 3, and 3, respectively. 

### 3.5. Codon Usage

To ensure sampling accuracy, only 52 PCGs with a length >300 bp were selected for synonymous codon usage analysis. Two relevant parameters, Nc and RSCU, were calculated using the program CodonW1.4.2 (Available online: http://downloads.fyxm.net/CodonW-76666.html). Nc is often utilized to evaluate the codon bias at the individual gene level, in a range from 20 (extremely biased) to 61 (totally unbiased) [58]. RSCU is the observed frequency of a codon divided by the expected frequency. The values close to 1.0 indicate a lack of bias [59]. AA frequency was also calculated and expressed by the percentage of the codons encoding the same amino acid divided by the total codons. 

### 3.6. Prediction of RNA Editing Sites

Prep-Cp [60] (Available online: http://prep.unl.edu/) and CURE software [61] (Available online: http://bioinfo.au.tsinghua.edu.cn/pure/) were applied to the prediction of RNA editing sites, and the parameter threshold (cutoff value) was set to 0.8 to ensure prediction accuracy.

### 3.7. Phylogenomic Analyses

ML phylogenetic analyses were performed using the *F. suspensa* complete cp genome and 32 Lamiales plastomes with three species, *Coffea arabica*, *Ipomoea purpurea*, and *Oryza nivara*, as outgroups (Table S1). All of the plastome sequences were aligned using MAFFT program version 7.0 [62] (Available online: http://mafft.cbrc.jp/alignment/server/index.html) and adjusted manually where necessary. These plastome nucleotide alignments were subjected to ML phylogenetic analyses with MEGA7.0 [63] based on the General Time Reversible model. A discrete Gamma distribution was used to model evolutionary rate differences among sites. The branch support was estimated by rapid bootstrap analyses using 100 pseudo-replicates.

## 4. Conclusions 

The cp genome of the medicinal plant *F. suspensa* was reported for the first time in this study and its organization is described and compared with that of other Lamiales species. This genome is 156,404 bp in length, with a similar quadripartite structure and genomic contents common to most land plant genomes. The low GC content of the cp genome might caused the codon usage bias toward A- or T-ending codons. All of the predicted RNA editing sites in the genome were C-to-U transitions. Among several relative species, the genome size and IR expansion or contraction exhibited some differences, and the divergent regions were also analyzed. Repeat sequences and SSRs within *F. suspensa* were analyzed, which may be useful in developing molecular markers for the analyses of infraspecific genetic differentiation within the genus *Forsythia* (Oleaceae). Phylogenetic analysis based on the entire cp genome revealed that *F. suspensa*, as a member of the Oleaceae family, diverged relatively early from Lamiales. Overall, the sequences and annotation of the *F. suspensa* cp genome will facilitate medicinal resource conservation, as well as molecular phylogenetic and genetic engineering research of this species.

## Figures and Tables

**Table 1:** A list of genes found in the plastid genome of *Forsythia suspensa.*

Category for Genes	Group of Gene	Name of Gene
Photosynthesis related genes	Rubisco	*rbcL*
	Photosystem І	*psaA*, *psaB*, *psaC*, *psaI*, *psaJ*
	Assembly/stability of photosystem І	ycf3 *, *ycf4*
	Photosystem ІІ	*psbA*, *psbB*, *psbC*, *psbD*, *psbE*, *psbF*, *psbH*, *psbI*, *psbJ*, *psbK*, *psbL*, *psbM*, *psbN*, *psbT*, *psbZ*
	ATP synthase	*atpA*, *atpB*, *atpE*, atpF *, *atpH*, *atpI*
	cytochrome b/f complex	*petA*, petB *, petD *, *petG*, *petL*, *petN*
	cytochrome c synthesis	*ccsA*
	NADPH dehydrogenase	ndhA *, ndhB *, *ndhC*, *ndhD*, *ndhE*, *ndhF*, *ndhG*, *ndhH*, *ndhI*, *ndhJ*
Transcription and translation related genes	transcription	*rpoA*, *rpoB*, rpoC1 *, *rpoC2*
	ribosomal proteins	*rps2*, *rps3*, *rps4*, *rps7*, *rps8*, *rps11*, rps12 *, *rps14*, *rps15*, rps16 *, *rps18*, *rps19*, rpl2 *, *rpl14*, rpl16 *, *rpl20*, *rpl22*, *rpl23*, *rpl32*, *rpl33*, *rpl36*
	translation initiation factor	*infA*
RNA genes	ribosomal RNA	*rrn5*, *rrn4.5*, *rrn16*, *rrn23*
	transfer RNA	trnA-UGC *, *trnC-GCA*, *trnD-GUC*, *trnE-UUC*, *trnF-GAA*, trnG-UCC *, trnG-GCC *, *trnH-GUG*, *trnI-CAU*, trnI-GAU *, trnK-UUU *, *trnL-CAA*, trnL-UAA *, *trnL-UAG*, *trnfM-CAUI*, *trnM-CAU*, *trnN-GUU*, *trnP-UGG*, *trnQ-UUG*, *trnR-ACG*, *trnR-UCU*, *trnS-GCU*, *trnS-GGA*, *trnS-UGA*, *trnT-GGU*, *trnT-UGU*, *trnV-GAC*, trnV-UAC *, *trnW-CCA*, *trnY-GUA*
Other genes	RNA processing	*matK*
	carbon metabolism	*cemA*
	fatty acid synthesis	*accD*
	proteolysis	clpP *
Genes of unknown function	conserved reading frames	*ycf1*, *ycf2*, *ycf15*, *ndhK*

* indicate the intron-containing genes.

**Table 2:** Genes with introns within the *F. suspensa* chloroplast genome and the length of exons and introns.

Gene	Location	Exon І (bp)	Intron І (bp)	Exon ІІ (bp)	Intron ІІ (bp)	Exon ІІІ (bp)
*trnA-UGC*	IR	38	814	35		
*trnG-GCC*	LSC	24	676	48		
*trnI-GAU*	IR	42	942	35		
*trnK-UUU*	LSC	38	2494	37		
*trnL-UAA*	LSC	37	473	50		
*trnV-UAC*	LSC	38	572	37		
*rps12* *	LSC	114	-	231	536	27
*rps16*	LSC	40	864	227		
*atpF*	LSC	144	705	411		
*rpoC1*	LSC	445	758	1619		
*ycf3*	LSC	129	714	228	737	153
*clpP*	LSC	69	815	291	642	228
*petB*	LSC	6	707	642		
*petD*	LSC	8	713	475		
*rpl16*	LSC	9	865	399		
*rpl2*	IR	393	664	435		
*ndhB*	IR	777	679	756		
*ndhA*	SSC	555	1106	531		

* The *rps12* is a trans-spliced gene with the 5′ end located in the LSC region and the duplicated 3′ end in the IR regions.

**Table 3:** The relative synonymous codon usage of the *Forsythia suspensa* chloroplast genome.

Amino Acids	Codon	Number	RSCU	AA Frequency	Amino Acids	Codon	Number	RSCU	AA Frequency
Phe	UUU	779	**1.32**	5.59%	Ser	UCU	472	**1.76**	7.59%
	UUC	405	0.68			UCC	247	0.92	
Leu	UUA	720	**1.93**	10.56%		UCA	307	**1.15**	
	UUG	451	**1.21**			UCG	152	0.57	
	CUU	486	**1.30**			AGU	339	**1.26**	
	CUC	129	0.35			AGC	91	0.34	
	CUA	301	0.81		Pro	CCU	351	**1.55**	4.26%
	CUG	150	0.40			CCC	170	0.75	
Ile	AUU	890	**1.47**	8.57%		CCA	269	**1.19**	
	AUC	377	0.62			CCG	113	0.50	
	AUA	548	0.91		Thr	ACU	430	**1.63**	4.98%
Met	AUG	495	1.00	2.34%		ACC	201	0.76	
Val	GUU	423	**1.48**	5.41%		ACA	324	**1.23**	
	GUC	126	0.44			ACG	100	0.38	
	GUA	447	**1.56**		Ala	GCU	526	**1.84**	5.41%
	GUG	151	0.53			GCC	177	0.62	
Tyr	UAU	631	**1.61**	3.70%		GCA	328	**1.14**	
	UAC	152	0.39			GCG	115	0.40	
TER	UAA	28	**1.62**	0.25%	Cys	UGU	171	**1.53**	1.05%
	UAG	10	0.58			UGC	52	0.47	
	UGA	14	0.81		Arg	CGU	275	**1.30**	6.00%
His	CAU	404	**1.58**	2.42%		CGC	90	0.42	
	CAC	108	0.42			CGA	284	**1.34**	
Gln	CAA	595	**1.52**	3.69%		CGG	97	0.46	
	CAG	186	0.48		Arg	AGA	392	**1.85**	
Asn	AAU	796	**1.56**	4.81%		AGG	133	0.63	
	AAC	224	0.44		Gly	GGU	493	**1.33**	7.00%
Lys	AAA	837	**1.54**	5.15%		GGC	145	0.39	
	AAG	253	0.46			GGA	594	**1.60**	
Asp	GAU	690	**1.59**	4.09%		GGG	251	0.68	
	GAC	176	0.41		Glu	GAA	866	**1.54**	5.32%
Trp	UGG	386	1.00	1.82%		GAG	262	0.46	

The value of relative synonymous codon usage (RSCU) > 1 are highlighted in bold.

**Table 4:** Repetitive sequences of *Forsythia suspensa* calculated using REPuter.

No.	Size/bp	Type ^#^	Repeat 1 Start (Location)	Repeat 2 Start (Location)	Region
1	30	F	10,814 (*trnG-GCC* *)	38,746 (*trnG-UCC*)	LSC
2	30	F	17,447 (*rps2-rpoC2*)	17,448 (*rps2-rpoC*)	LSC
3	30	F	44,547 (*psaA-ycf3*)	44,550 (*psaA-ycf3*)	LSC
4	30	F	45,978 (*ycf3* intron2)	101,338 (*rps12_3end-trnV-GAC*)	LSC, IRa
5	30	F	91,923 (*ycf2*)	91,965 (*ycf2*)	IRa
6	30	F	110,167 (*rrn4.5-rrn5*)	110,198 (*rrn4.5-rrn5*)	IRa
7	30	F	133,335 (*rrn5-rrn4.5*)	133,366 (*rrn5-rrn4.5*)	IRb
8	30	F	149,178 (*ycf2*)	149,214 (*ycf2*)	IRb
9	30	F	149,196 (*ycf2*)	149,214 (*ycf2*)	IRb
10	30	F	151,568 (*ycf2*)	151,610 (*ycf2*)	IRb
11	32	F	9313 (*trnS-GCU* *)	37,781 (*psbC-trnS-UGA* *)	LSC
12	32	F	40,965 (*psaB*)	43,189 (*psaA*)	LSC
13	32	F	53,338 (*ndhC-trnV-UAC*)	53,358 (*ndhC-trnV-UAC*)	LSC
14	32	F	115,350 (*ndhF-rpl32*)	115,378 (*ndhF-rpl32*)	SSC
15	34	F	94,332 (*ycf2*)	94,368 (*ycf2*)	IRa
16	34	F	94,350 (*ycf2*)	94,368 (*ycf2*)	IRa
17	35	F	149,188(*ycf2*)	149,206 (*ycf2*)	IRb
18	39	F	45,966 (*ycf3* intron2)	101,326 (*rps12_3end-trnV-GAC*)	LSC, IRa
19	39	F	45,966 (*ycf3* intron2)	122,604 (*ndhA* intron1)	LSC, SSC
20	41	F	40,953 (*psaB*)	43,177 (*psaA*)	LSC
21	41	F	101,324 (*rps12_3end-trnV-GAC*)	122,602 (*ndhA* intron)	IRa, SSC
22	42	F	94,320 (*ycf2*)	94,356 (*ycf2*)	IRa
23	42	F	149,165 (*ycf2*)	149,201 (*ycf2*)	IRb
24	44	F	94,340 (*ycf2*)	94,358 (*ycf2*)	IRa
25	58	F	94,332 (*ycf2*)	94,340 (*ycf2*)	IRa
26	58	F	149,165 (*ycf2*)	149,183 (*ycf2*)	IRb
27	30	P	9315 (*trnS-GCU* *)	47,653 (*trnS-GGA*)	LSC
28	30	P	14,359 (*atpF-atpH*)	14,359 (*atpF-atpH*)	LSC
29	30	P	34,338 (*trnT-GGU-psbD*)	34,338 (*trnT-GGU-psbD*)	LSC
30	30	P	37,783 (*psbC-trnS-UGA* *)	47,653 (*trnS-GGA*)	LSC
31	30	P	45,978 (*ycf3* intron2)	142,195 (*trnV-GAC-rps12_3end*)	LSC, IRb
32	30	P	91,923 (*ycf2*)	151,568 (*ycf2*)	IRa, IRb
33	30	P	91,965 (*ycf2*)	151,610 (*ycf2*)	IRa, IRb
34	30	P	110,167 (*rrn4.5-rrn5*)	133,335 (*rrn5-rrn4.5*)	IRa, IRb
35	30	P	110,198 (*rrn4.5-rrn5*)	133,366 (*rrn5-rrn4.5*)	IRa, IRb
36	30	P	122,764 (*ndhA* intron1)	122,766 (*ndhA* intron1)	SSC
37	34	P	94,332 (*ycf2*)	149,161 (*ycf2*)	IRa, IRb
38	34	P	94,350 (*ycf2*)	149,161 (*ycf2*)	IRa, IRb
39	34	P	94,368 (*ycf2*)	149,179 (*ycf2*)	IRa, IRb
40	34	P	94,368 (*ycf2*)	149,179 (*ycf2*)	IRa, IRb
41	39	P	45,966 (*ycf3* intron2)	45,966 (*ycf3* intron2)	LSC, IRb
42	41	P	122,602 (*ndhA* intron1)	142,198 (*trnV-GAC–rps12_3end*)	SSC, IRb
43	42	P	94,320 (*ycf2*)	149,165 (*ycf2*)	IRa, IRb
44	42	P	94,356 (*ycf2*)	149,201 (*ycf2*)	IRa, IRb
45	44	P	77,475 (*psbT-psbN*)	77,475 (*psbT-psbN*)	LSC
46	44	P	94,340 (*ycf2*)	149,161 (*ycf2*)	IRa, IRb
47	44	P	94,358 (*ycf2*)	149,179 (*ycf2*)	IRa, IRb
48	58	P	94,332 (*ycf2*)	149,165 (*ycf2*)	IRa, IRb
49	58	P	94,340 (*ycf2*)	149,183 (*ycf2*)	IRa, IRb

^#^ F: forward; P: palindrome; * part in the gene.

**Table 5:** Distribution of SSR loci in the chloroplast genome of *Forsythia suspensa*.

SSR Type ^#^	SSR Sequence	Size	Start	SSR Location	Region
p1	(A)10	10	31,855	*psbM-trnD-GUC*	LSC
		10	31,992	*psbM-trnD-GUC*	LSC
		10	38,025	*trnS-UGA-psbZ*	LSC
		10	73,886	*clpP* intron1	LSC
		10	85,390	*rpl16* intron	LSC
	(T)10	10	507	*trnH-GUG-psbA*	LSC
		10	9056	*psbK-psbI*	LSC
		10	11,162	*trnR-UCU-atpA*	LSC
		10	59,781	*rbcL-accD*	LSC
		10	66,291	*petA-psbJ*	LSC
		10	69,202	*petL-petG*	LSC
	(C)10	10	5236	*trnK-UUU-rps16*	LSC
	(T)11	11	19,678	*rpoC2*	LSC
		11	50,871	*trnF-GAA-ndhJ*	LSC
		11	61,662	*accD-psaI*	LSC
		11	72,263	*rpl20-clpP*	LSC
		11	74,741	*clpP* intron2	LSC
	(T)12	12	20,216	*rpoC2*	LSC
		12	81,254	*rpoA*	LSC
		12	83,666	*rps8-rpl14*	LSC
	(A)13	13	12,741	*atpA-atpF*	LSC
		13	46,877	*ycf3-trnS-GGA*	LSC
	(T)13	13	14,109	*atpF-atpH*	LSC
		13	34,486	*trnT-GGU-psbD*	LSC
		13	37,645	*psbC-trnS-UGA*	LSC
		13	86,860	*rpl22-rps19*	LSC
	(T)14	14	48,630	*rps4-trnT-UGU*	LSC
	(A)15	15	33,163	*trnE-UUC-trnT-GGU*	LSC
	(A)16	16	46,618	*ycf3* intron2	LSC
	(A)19	19	44,559	*psaA-ycf3*	LSC
	(T)19	19	117,928	*ndhD*	SSC
	(A)20	20	29,957	*trnC-GCA-petN*	LSC
p2	(AT)5	10	4646	*trnK-UUU-rps16*	LSC
		10	6558	*rps16-trnQ-UUG*	LSC
		10	21,057	*rpoC2*	LSC
	(TA)5	10	69,619	*trnW-CCA-trnP-UGG*	LSC
	(TA)6	12	48,772	*rps4-trnT-UGU*	LSC
		12	49,291	*trnT-UGU-trnL-UAA*	LSC
		12	69,931	*trnP-UGG-psaJ*	LSC
p3	(CCT)4	12	69,371	*petG-trnW-CCA*	LSC
p4	(AAAG)3	12	73,413	*clpP* intron1	LSC
	(TCTT)3	12	31,191	*petN-psbM*	LSC
	(TTTA)3	12	55,102	*trnM-CAU-atpE*	LSC
	(AAAT)4	16	9284	*psbI-trnS-GCU*	LSC
p5	(TCTAT)3	15	9458	*trnS-GCU-trnG-GCC*	LSC
c	-	23	17,456	*rps2-rpoC2*	LSC
	-	27	63,589	*ycf4-cemA*	LSC
	-	33	78,324	*petB* intron	LSC
	-	45	71,570	*rps18-rpl20*	LSC
	-	59	38,501	*psbZ-trnG-UCC*	LSC
	-	90	57,078	atpB *	LSC

^#^ p1: mono-nucleotide; p2: di-nucleotide; p3: tri-nucleotide; p4: tetra-nucleotide; p5: penta-nucleotide; c: compound; * part in the gene.

**Table 6:** The predicted RNA editing site in the *Forsythia suspensa* chloroplast genes.

Gene	Codon Position	Amino Acid Position	Codon (Amino Acid) Conversion	Score
*accD*	794	265	uCg (S) => uUg (L)	0.8
	1403	468	cCu (P) => cUu (L)	1
*atpA*	914	305	uCa (S) => uUa (L)	1
*atpF*	92	31	cCa (P) => cUa(L)	0.86
*atpI*	629	210	uCa (S) => uUa (L)	1
*ccsA*	71	24	aCu (T) => aUu (I)	1
*matK*	271	91	Ccu (P) => Ucu (S)	0.86
	460	154	Cac (H) => Uac (Y)	1
	646	216	Cau (H) => Uau (Y)	1
	1180	394	Cgg (R) => Ugg (W)	1
	1249	417	Cau (H) => Uau (Y)	1
*ndhA*	344	115	uCa (S) => uUa (L)	1
	569	190	uCa (S) => uUa (L)	1
*ndhB*	149	50	uCa (S) => uUa (L)	1
	467	156	cCa (P) => cUa (L)	1
	586	196	Cau (H) => Uau (Y)	1
	611	204	uCa (S) => uUa (L)	0.8
	737	246	cCa (P) => cUa (L)	1
	746	249	uCu (S) => uUu (F)	1
	830	277	uCa (S) => uUa (L)	1
	836	279	uCa (S) => uUa (L)	1
	1292	431	uCc (S) => uUc (F)	1
	1481	494	cCa (P) => cUa (L)	1
*ndhD*	2	1	aCg (T) => aUg (M)	1
	47	16	uCu (S) => uUu (F)	0.8
	313	105	Cgg (R) => Ugg (W)	0.8
	878	293	uCa (S) => uUa (L)	1
	1298	433	uCa (S) => uUa (L)	0.8
	1310	437	uCa (S) => uUa (L)	0.8
*ndhF*	290	97	uCa (S) => uUa (L)	1
	671	224	uCa (S) => uUa (L)	1
*ndhG*	314	105	aCa (T) => aUa (I)	0.8
	385	129	Cca (P) => Uca (S)	0.8
*petB*	418	140	Cgg (R) => Ugg (W)	1
	611	204	cCa (P) => cUa (L)	1
*petG*	94	32	Cuu (L) => Uuu (F)	0.86
*psbE*	214	72	Ccu (P) => Ucu (S)	1
*rpl2*	596	199	gCg (A) => gUg (V)	0.86
*rpl20*	308	103	uCa (S) => uUa (L)	0.86
*rpoA*	830	277	uCa (S) => uUa (L)	1
*rpoB*	338	113	uCu (S) => uUu (F)	1
	551	184	uCa (S) => uUa (L)	1
	566	189	uCg (S) => uUg (L)	1
	1672	558	Ccc (P) => Ucc (S)	0.86
	2000	667	uCu (S) => uUu (F)	1
	2426	809	uCa (S) => uUa (L)	0.86
*rpoC2*	1792	598	Cgu (R) => Ugu (C)	0.86
	2305	769	Cgg (R) => Ugg (W)	1
	3746	1249	uCa (S) => uUa (L)	0.86
*rps2*	248	83	uCa (S) => uUa (L)	1
*rps14*	80	27	uCa (S) => uUa (L)	1
	149	50	cCa (P) => cUa (L)	1

## Supplementary Materials

Supplementary materials can be found at www.mdpi.com/1422-0067/18/11/2288/s1.
