# Supplementary material for: The Complete Chloroplast Genome Sequences of the Medicinal Plant Forsythia suspensa (Oleaceae)
Source: Int J Mol Sci. 2017 Oct 31;18(11):2288. doi: 10.3390/ijms18112288 (PMC5713258; doi:10.3390/ijms18112288)
Supplement: Supplementary file 1 [file ijms-18-02288-s001.zip › ijms-228837 supplementary/S1. Accession numbers of the chloroplast genomes sequences used in this study.pdf]

Table S1. Accession numbers of the chloroplast genome sequences used in this study

NO.	Taxon	Order	Family	GenBank Accession Number
1	*Forsythia suspensa*	Lamiales	Oleaceae	MF579702
2	*Olea europaea*	Lamiales	Oleaceae	NC_015604.1
3	*Olea woodiana*	Lamiales	Oleaceae	NC_015608.1
4	*Hesperelaea palmeri*	Lamiales	Oleaceae	NC_025787.1
5	*Abeliophyllum distichum*	Lamiales	Oleaceae	NC_031445.1
6	*Jasminum nudiflorum*	Lamiales	Oleaceae	NC_008407.1
7	*Perilla setoyensis*	Lamiales	Lamiaceae	NC_030757.1
8	*Perilla frutescens*	Lamiales	Lamiaceae	NC_030756.1
9	*Perilla citriodora*	Lamiales	Lamiaceae	NC_030755.1
10	*Stachys byzantina*	Lamiales	Lamiaceae	NC_029825.1
11	*Stachys sylvatica*	Lamiales	Lamiaceae	NC_029824.1
12	*Stenogyne kanehoana*	Lamiales	Lamiaceae	NC_029821.1
13	*Stenogyne bifida*	Lamiales	Lamiaceae	NC_029818.1
14	*Salvia miltiorrhiza*	Lamiales	Lamiaceae	NC_020431.1
15	*Salvia rosmarinus*	Lamiales	Lamiaceae	NC_027259.1
16	*Pogostemon stellatus*	Lamiales	Lamiaceae	NC_031434.1
17	*Pogostemon yatabeanus*	Lamiales	Lamiaceae	NC_031433.1
18	*Scutellaria insignis*	Lamiales	Lamiaceae	NC_028533.1
19	*Scutellaria baicalensis*	Lamiales	Lamiaceae	NC_027262.1
20	*Rehmannia chingii*	Lamiales	Orobanchaceae	NC_033534.1
21	*Pedicularis ishidoyana*	Lamiales	Orobanchaceae	NC_029700.1
22	*Utricularia macrorhiza*	Lamiales	Lentibulariaceae	NC_025653.1
23	*Utricularia gibba*	Lamiales	Lentibulariaceae	NC_021449.1
24	*Genlisea margaretae*	Lamiales	Lentibulariaceae	NC_025652.1
25	*Scrophularia takesimensis*	Lamiales	Scrophulariaceae	KP718628.1
26	*Scrophularia buergeriana*	Lamiales	Scrophulariaceae	NC_031437.1
27	*Scrophularia dentata*	Lamiales	Scrophulariaceae	KT428154.1
28	*Boea hygrometrica*	Lamiales	Gesneriaceae	NC_016468.1
29	*Haberlea rhodopensis*	Lamiales	Gesneriaceae	NC_031852.1
30	*Paulownia coreana*	Lamiales	Paulowniaceae	NC_031435.1
31	*Paulownia tomentosa*	Lamiales	Paulowniaceae	NC_031436.1
32	*Sesamum indicum* L. cv. Ansanggae	Lamiales	Pedaliaceae	JN637766.2
33	*Sesamum indicum* L. cv. Yuzhi 11	Lamiales	Pedaliaceae	KC569603.1
34	*Tanaecium tetragonolobum*	Lamiales	Bignoniaceae	NC_027955.1
35	*Erythranthe lutea*	Lamiales	Phrymaceae	NC_030212.1
36	*Andrographis paniculata*	Lamiales	Acanthaceae	NC_022451.2
37	*Coffea arabica*	Gentianales	Rubiaceae	NC_008535
38	*Ipomoea purpurea*	Solanales	Convolvulaceae	NC_009808
39	*Oryza nivara*	Poales	Poaceae	NC_005973
